# Fabrication of Novel and Potential Selective 4-Cyanophenol Chemical Sensor Probe Based on Cu-Doped Gd_2_O_3_ Nanofiber Materials Modified PEDOT:PSS Polymer Mixtures with Au/µ-Chip for Effective Monitoring of Environmental Contaminants from Various Water Samples

**DOI:** 10.3390/polym13193379

**Published:** 2021-09-30

**Authors:** Mohammed Muzibur Rahman, S. Y. Alfaifi

**Affiliations:** 1 Department of Chemistry, Faculty of Science, King Abdulaziz University, P.O. Box 80203, Jeddah 21589, Saudi Arabia; syalfaifikau@gmail.com; 2 Center of Excellence for Advanced Materials Research (CEAMR), King Abdulaziz University, P.O. Box 80203, Jeddah 21589, Saudi Arabia

**Keywords:** Cu-doped Gd_2_O_3_ nanofibers, tiny Au/µ-Chip, 4-Cyanophenol, PEDOT:PSS polymer matrixes, real sample analysis, electrochemical method

## Abstract

Herein, a novel copper-doped gadolinium oxide (Cu-doped Gd_2_O_3_; CGO) nanofiber was synthesized by a simple solution method in the basic phase and successfully characterized. We have used Fourier Transform Infrared Spectroscopy (FTIR), X-ray Diffraction (XRD), Field Emission Scanning Electron Microscope (FESEM) and Energy-Dispersive Spectroscopy (EDS) techniques for characterization of the CGO nanofiber. The CGO nanofiber was used later to modify Au-coated μ-Chips with poly(3,4-ethylenedioxythiophene) polystyrene sulfonate (PEDOT:PSS) polymer mixtures (coating binder) to selectively detect 4-cyanophenol (4-CP) in an aqueous medium. Notable sensing performance was achieved with excellent sensitivity (2.4214 µAµM^−1^ cm^−2^), fast response time (~12 s), wide linear dynamic range (LDR = 1.0 nM–1.0 mM: R^2^ = 0.9992), ultra-low detection limit (LoD; 1.3 ± 0.1 pM at S/N = 3), limit of quantification (LoQ; 4.33 pM), and excellent reproducibility and repeatability for CGO/Au/μ-Chip sensor. This CGO modified Au/μ-chip was further applied with appropriate quantification and determination results in real environmental sample analyses.

## 1. Introduction

Generally, phenol derivatives are usually toxic for human beings and aquatic animals, even at trace levels [1,2,3]. The main source of these environmental pollutants in surface water are industrial effluents, domestic discharge, usage of pesticides, and automobile exhaust [4,5,6]. 4-cyanophenol (4-CP), a widely used phenol derivative, is considered as an environmental toxin posing a health hazard [7,8]. United States Environmental Protection Agency (US EPA) also declared this 4-CP as a major environmental pollutant [9,10]. 4-CP is also commonly used as an antifungal [11], insecticidal [12] agents and its adverse effect has been studied by researchers [8,13,14]. Inhalation of 4-CP can harm the central nervous system and disturb the cardiac systems [15]. 4-CP might get metabolized in the human body yielding toxic cyanide which can cause severe respiratory problems [16,17]. The cyanide formation might also cause headache, dizziness, and unconsciousness, and occasionally even death [13]. Upon contact, it causes eye, nose, skin, and respiratory system irritation. 4-CP is also harmful to fish [18]. Therefore, an effective determination of 4-CP becomes necessary [19]. Several techniques have been used to detect 4-CP such as UV-Vis spectroscopy, gas chromatography, and capillary electrophoresis [13,20,21]. However, the electrochemical techniques are advantageous in terms of sensitivity, low-cost, response-time, and pretreatment of samples not required so suitable for on-field detection [22,23,24,25,26]. Thus electrochemical techniques gain substantial consideration in recent years [27,28,29,30,31,32]. Still, interference from similar chemicals is often observed in spectrophotometric techniques. While a huge amount of ultrapure organic solvents are required for chromatographic techniques and GC-MS. Moreover, these are slow and expensive and, therefore, not suitable for on-field detection. These prevailing methods are costly and often involve difficult phases, and hence are extremely time-consuming. Due to reduced sensitivity and selectivity, these methods become unsuitable for routine detection of 4-CP. Regrettably, direct 4-CP detection using bare electrodes such as GCE, platinum, and gold electrodes is challenging because of the reduced responses. Moreover, a bare electrode frequently suffers from over-potential. Therefore, the development of new active materials material for electrode modification becomes important.

Lately, the electrochemical detection of hazardous chemicals by chemically modified electrodes (CMEs) has become vital due to their quick response, cheap method, handy nature, and high sensitivity, especially in situ detection [33,34]. Developing an active material with better electro-catalytic activity and superior conductivity is the key feature in CMEs. Recently, modification of an electrode by nanomaterials such as transition metal oxides, sulphides, or various types of nanocomposites (NCs) becomes an interesting research topic [35]. Scientists have explored thin films consisting of composite of various transition-metal-doped rare earth metal oxides to detect pollutants. Of these metal oxides, gadolinium oxide (Gd_2_O_3_) is an interesting material for sensing since it gives a suitable environment for doping elements as a host because of its high band-gap, low phonon frequency, and good thermal and chemical stability [36,37,38]. In recent years, studies on doped and undoped Gd_2_O_3_ nanoparticles focus on their luminescence properties, but herein we have investigated Cu-doped Gd_2_O_3_. The incorporation of transition metals may affect the structural and optical properties of the materials. So far, several phosphors have been reported via doping of copper and lanthanide combinations, but no such report is available with Cu-doped Gd_2_O_3_. Therefore, it is interesting to see the effect of the incorporation of CuO into the Gd_2_O_3_.

**Scheme 1:**
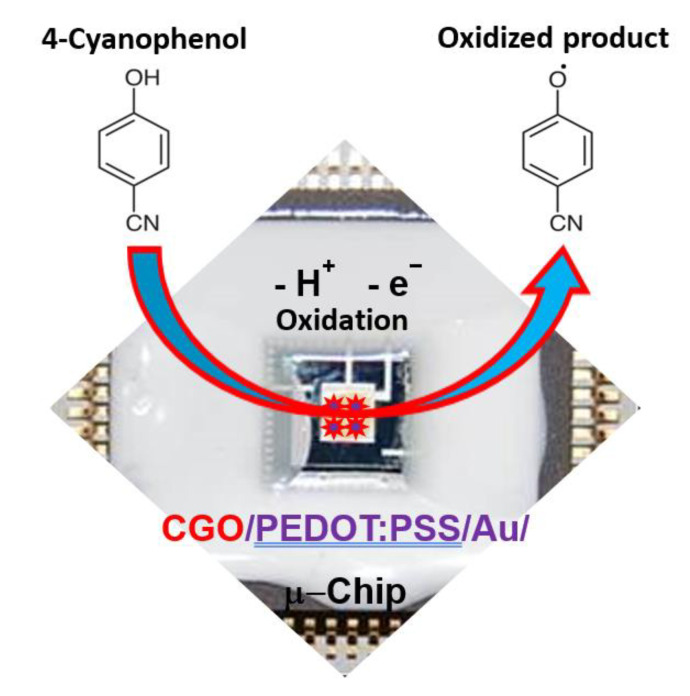
Schematic representation of 4-CP detection with CGO/PEDOT:PSS/Au/μ-Chip.

Herein, we have reported the synthesis and systematic characterization of the CGO. Additionally, a micro-chip was fabricated by CGO using the PEDOT:PSS in developing a sensor to detect 4-CP, which is presented in the Scheme 1. A simple I-V method at ambient conditions was used in this study since it is handy, low-cost, and less solvent required thus green. 4-CP is oxidized onto CGO/PEDOT:PSS/Au/μ-Chip in electrochemical process to release electrode and form oxidized production during electrochemical process in a room conditions. To the best of our knowledge, it would be the first report of selective 4-CP sensor probe fabrication based on oxidation mechanism by using the formulated CGO as the active nanostructure material embedded tiny Au/μ-chip by using PEDOT:PSS polymer mixtures.

## 2. Experimental

### 2.1. Materials and Methods

Hydrated copper (II) chloride, Gadolinium (III) chloride, ammonium hydroxide, 4-Cyanophenol, PEDOT:PSS (poly(3,4-ethylenedioxythiophene) polystyrene sulfonate), dopamine, Catechol, ascorbic acid, uric acid, 4-nitrophenol, hydroquinone, ethanol, hydrazine, 4-cyanophenol, etc., used in the current study were from Sigma-Aldrich, Burlington, MA, USA and all of them were used as received. For CGO nanofiber an FTIR spectrum was studied by NICOLET iS50 FTIR spectrometer, Thermo Scientific, Waltham, MA, USA). The powder XRD prototypes of the CGO nanofiber was studied by the X-ray diffractometer (XRD, Thermo scientific, ARL X’TRA diffractometer, Waltham, MA, USA). The morphology of CGO nanofiber was studied by the FESEM (JEOL, JSM-7600 F, Tokyo, Japan). The elemental analysis was performed by the EDS from JEOL, Tokyo, Japan. I-V method was used by the Keithley, 6517A Electrometer (Solon, OH, USA) at the normal temperature.

### 2.2. Synthesis of the CGO Nanofibers 

We have synthesized the CGO nanofibers by a simple solution method. Briefly, in this reaction, equimolar (0.1 M) Cu^2+^, Gd^3+^, and NH_4_OH have been taken. These ions were mixed (50 mL each) in a 250 mL conical flask for half an hour with continuous stirring at 60 °C. Then, we have added 100 mL of aqueous NH_4_OH (0.1 M) dropwise to this mixture with constant stirring. Continued the stirring for 6 h at 70 °C. On cooling, a gray-precipitate of CGO nanofiber was produced. It is later washed with double distilled water and ethanol. Then, we dried this precipitate at ambient conditions for half an hour. After that, we heated the precipitate for 2 h at 65 °C to get the as-grown CGO nanofiber. We then heated the as-grown CGO nanofiber for 5 h at 500 °C to convert it to the CGO nanofiber. 

### 2.3. Fabrication of Au/µ-Chip by CGO Nanofibers with PEDOT:PSS

In this approach, modification of gold-coated micro-Chip was performed by the CGO nanofiber by using PEDOT:PSS. A total of 1.0 mg of CGO was taken initially onto the watch-glass. Then, 1.0 uL of PEDOT:PSS was placed onto the CGO and mixed properly. The mixture was deposited onto the micro-chip. Then, the fabricated gold-coated μ-Chip was dried in ambient conditions for 1 h to obtain a thin film on the gold-coated μ-Chip for CGO/PEDOT:PSS/Au/µ-Chip. In an electrochemical cell, a fabricated CGO/PEDOT:PSS/Au/μ-Chip, Pt-line, and 4-CP solution in PBS (pH 7.0) were used as a working electrode (WE), a counter electrode (CE), and target analyte, respectively. 4-CP solution (0.1 M) was taken as a stock solution for the targeted analyte and electrochemical methods were engaged to detect 4-CP.

## 3. Results and Discussion

### 3.1. Characterization of the CGO Nanofibers

**Figure 1:**
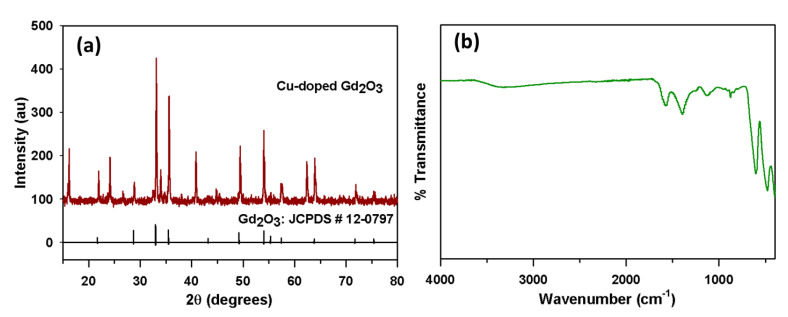
Structural and Optical characterization. (**a**) XRD spectrum and (**b**) FTIR spectrum of CGO nanofiber.

The XRD patterns of the CGO nanofiber are displayed in Figure 1a. This pattern confirmed the presence of the Gd_2_O_3_ cubic phase. The diffraction peaks appeared at 2*θ* values of 21.7°, 28.7°, 33.0°, 35.5°, 43.2°, 49.2°, 54.0°, 55.3°, 57.4°, 63.8°, 71.7°, and 75.4° can be correlated to the planes (211), (222), (400), (411), (431), (440), (541), (622), (631), (444), (622), (811), and (662) of the cubic Gd_2_O_3_ with space group of Ia3 (JCPDS # 12-0797 and 88-2165) [39,40,41,42,43]. While diffraction peaks appeared at 2*θ* value 33.9°, 62.3°, and 65.6° can be assigned to (111)), (113), and (022) planes of cubic CuO, respectively [44,45,46,47]. These XRD peaks can be assigned to the standard Cu doped Gd_2_O_3_ cubic crystal phase. The EDS results also showed that the as-grown CGO consists of Gd, Cu, and O with a respective weight percentage of 94.25%, 2.17%, and 3.58%.

The CGO nanofiber was further studied by FTIR to find out their atomic vibrations as in Figure 1b. The Gd_2_O_3_ displays an absorption band at 565 cm^−1^ in accordance with the metal-oxygen vibrational mode of absorption, which is just matched with literature values [48,49]. The absorption band that appears at 1578 cm^−1^ are because of the overtone of Gd_2_O_3_. The absorption bands appeared at 481, and 880, cm^−1^ are due to vibration between Cu and O atoms [50]. The absorption bands at 1127 and 1410 cm^−1^ are because of the vibrational overtone. 

**Figure 2:**
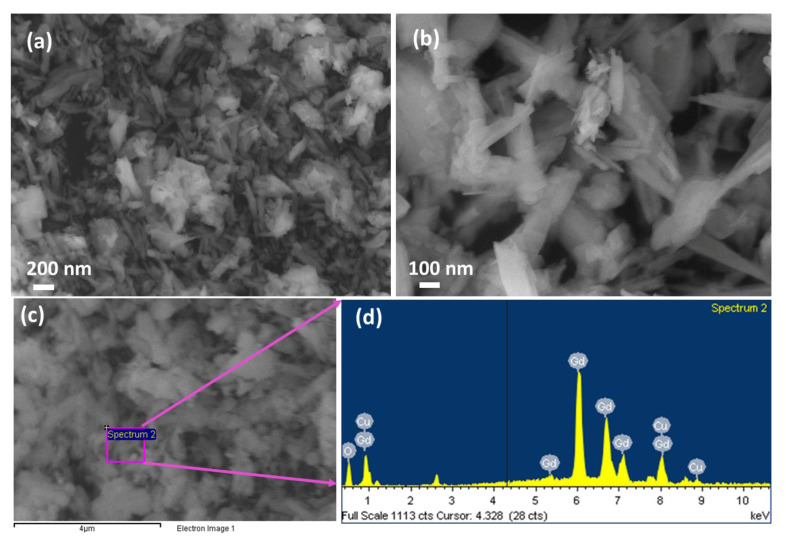
Morphological and elemental analysis. (**a**,**b**) Low to high-resolution FE-SEM images and (**c**,**d**) EDS spectrum of the CGO nanofibers.

The morphological and surface structure of the CGO nanofiber was explored by FESEM (Figure 2a,b). The CGO nanofiber is consisting of Cu-doped Gd_2_O_3_ that has fiber-like aggregated morphological structure with nano-size distributions. The diameter of CGO fibers is in the range of 30 to 150 nm. FESEM image showed the average nanofiber of CGO having a mean diameter of ~45 nm. The elemental composition of CGO nanofiber was studied by EDS (Figure 2c,d), which indicates that this CGO nanofiber consists of Gd, Cu and O with a respective weight percentage of 94.25%, 2.17%, and 3.58%.

### 3.2. 4-Cyanophenol Sensor Development

#### 3.2.1. Detection of 4-CP Using the CGO/Au/μ-Chip

Toxic 4-CP from the aqueous solution was detected by the CGO modified gold-coated μ-Chip as an electrochemical sensor. During electrochemical measurements, 4-CP produced a significant response as 4-CP touches the CGO/PEDOT:PSS/Au/μ-Chip. Therefore, we have proposed a 4-CP sensor based on the CGO/PEDOT:PSS/Au/μ-Chip assembly in phosphate-buffered solution (PBS). This will be the maiden 4-CP sensor based on the CGO/PEDOT:PSS/Au/μ-Chip assembly. 

**Scheme 2:**
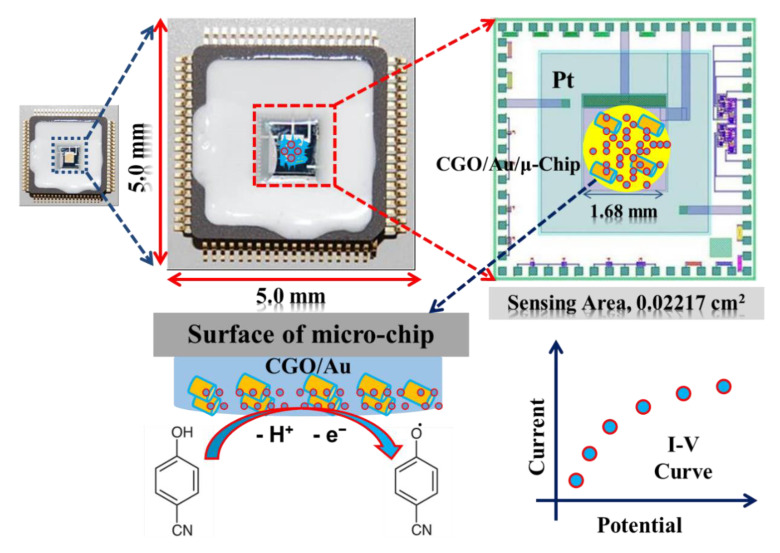
Proposed electrochemical oxidation mechanism of 4-CP on CGO/PEDOT:PSS/Au/μ-Chip assembly.

Here, the tiny microchip is fabricated with the prepared CGO with the polymer mixtures of PEDOT:PSS. In electrochemical detection, the current response was increased remarkably in presence of the 4-CP concentration on the surface of fabricated microchip. In the electrochemical oxidation of 4-CP at the CGO/PEDOT:PSS/Au/μ-Chip assembly, one electron and one proton were transferred to the conduction band of the CGO/PEDOT:PSS/Au/μ-Chip assembly by 4-CP [21], which causes the increasing of resultant current responses as shown in Scheme 2. The fabrication procedure and detection mechanism are schematically presented here.

Herein, a gold microchip was modified with CGO nanofiber using PEDOT:PSS and dried at ambient conditions for 2 h. Then, the modified CGO/PEDOT:PSS/Au/μ-Chip was used in detecting 4-CP. The 4-CP oxidation at CGO/PEDOT:PSS/Au/μ-Chip in PBS is recommended as in Equation (1). In the electrochemical oxidation process, target 4-CP molecules were oxidized by losing one electron at the conduction-band of the CGO/PEDOT:PSS/Au/μ-Chip hence increases the current response. Thus, when 4-CP molecule comes in contact with the CGO/PEDOT:PSS/Au/μ-Chip surface, 4-CP molecules were oxidized by releasing one electron and one proton on the sensor CGO/PEDOT:PSS/Au/μ-Chip surface.
(1)
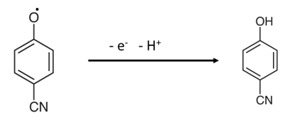


**Figure 3:**
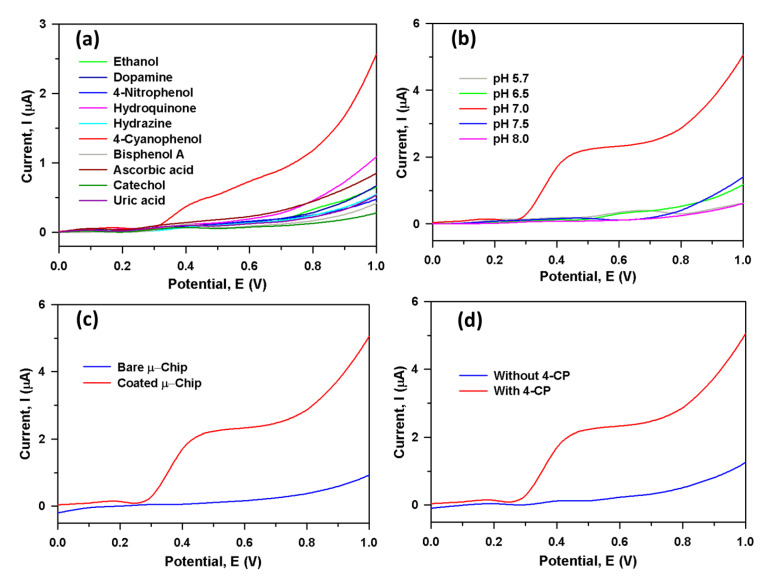
Current response from 2.0 µM; 25.0 µL using the CGO/PEDOT:PSS/Au/μ-Chip electrode: (**a**) Selectivity study of ten interfering chemicals, (**b**) pH optimization, (**c**) Au/μ-Chip and CGO/PEDOT:PSS/Au/μ-Chip electrode and (**d**) Without and with the presence of 4-CP.

Figure 3a displayed the current responses for ten toxic interfering chemicals in the selectivity study, where aqueous 4-CP (red line) in PBS gave a distinguishable higher current response at CGO/Au/μ-Chip assembly. Because of the ability to distinguish interfering agents from the 4-CP with very close electrochemical behavior, the interference study is one of the important methods of analytical chemistry, particularly transition metal-doped semiconductor metal oxide-based sensor probes. To study the effect of various interfering chemicals, the modified electrode was examined to check the acceptance in ideal conditions in 5.0 µM 4-CP. From where, it can get the highest concentrations of interfering substances that cause no more than 5% error. Thus, these electrochemical study reveals that equal concentration of hydrazine, 4-nitophenol, hydroquinone, ethanol, bisphenol A, catechol, ascorbic acid, uric acid, and dopamine showed a negligible effect on the current response of 4-CP. Therefore, it confirmed that the CGO/PEDOT:PSS/Au/μ-Chip assembly is selective towards the 4-CP in the presence of above-mentioned interfering chemicals. The proposed CGO/PEDOT:PSS/Au/μ-Chip probe is appropriate in the determination of 4-CP with high sensitivity. We have also studied the pH effect of CGO/PEDOT:PSS/Au/μ-Chip towards 4-CP for different pH values ranging from 5.7–8.0 Figure 3b. From the experiments, it is clear that the CGO/PEDOT:PSS/Au/μ-Chip displays good electrocatalytic activities at various pH values. In Figure 3b, the pH effect study with 4-CP reveals that at 7.0 pH (red line), the highest current output was observed. Therefore, pH ~7.0 was kept constant for all other experiments in the 4-CP detection with CGO/PEDOT:PSS/Au/μ-Chip sensor. Figure 3c exhibits the current responses from 4-CP in PBS at bare Au-coated μ-Chip (blue line) and CGO/PEDOT:PSS/Au/μ-Chip (red line). The CGO/PEDOT:PSS/Au/μ-Chip made a considerably improved response compared to a gold-coated μ-Chip electrode, which confirmed the exceptional electrochemical property of CGO/PEDOT:PSS/Au/μ-Chip towards 4-CP at ambient conditions. Figure 3d displays the current output from the CGO/PEDOT:PSS/Au/μ-Chip with 4-CP (red line) and in the absence of 4-CP (blue line). With 4-CP, a substantial upsurge of output current specifies the 4-CP sensing ability of the CGO/PEDOT:PSS/Au/μ-Chip at ambient conditions.

**Figure 4:**
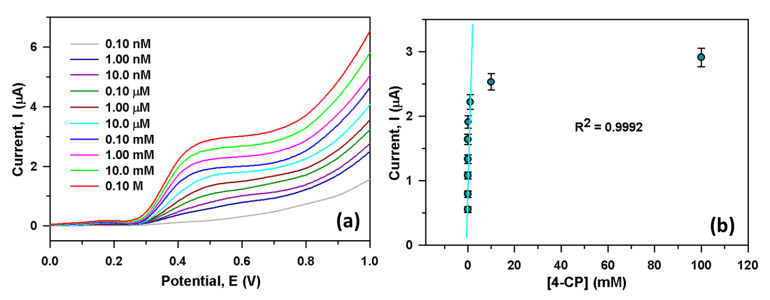
(**a**) Electrochemical responses for different 4-CP solution (0.10 nM to 0.10 M) and (**b**) Calibration curve of the CGO/Au/μ-Chip at +0.5 V.

We have sequentially injected 25.0 µL 4-CP (0.10 nM to 0.10 M) in 5.0 mL PBS; then, the variation of current response was investigated for each injection. Figure 4a showed the current responses from the CGO/PEDOT:PSS/Au/μ-Chip for various 4-CP solutions (0.10 nM to 0.10 M). It showed that output current rises for CGO/PEDOT:PSS/Au/μ-Chip probe while 4-CP concentration increases. It was also seen that from a lower (0.10 nM) to a higher concentration (0.10 M) of 4-CP, the output current also rises gradually. A varying concentration of 4-CP (0.10 nM to 0.10 M) was used to select the LOD and LDR of the newly developed 4-CP sensor. Figure 4b shows the calibration plot at +0.5 V, from which extremely high sensitivity value was estimated as 2.4214 µAµM^−1^cm^−2^, while the LDR of the developed CGO/PEDOT:PSS/Au/μ-Chip assembly was attained as 1.0 nM to 1.0 mM (R^2^ = 0.9992). An ultra-low LOD value was also obtained as 1.3 ± 0.1 pM (3 × N/S).

Excellent reproducibility was achieved using five different CGO/PEDOT:PSS/Au/μ-Chip electrodes under identical conditions, resulting in a relative standard deviation (RSD) of ~3.6%. The CGO/PEDOT:PSS/Au/μ-Chip sensor’s repeatability was also tested for seven successive runs in 2.0 μM 4-CP, resulting in a current variance of RSD ~4.1%. After 28 days of electrode storage under room conditions a nominal decrease in sensitivity was observed, all of which are useful in the practical use of this sensor.

#### 3.2.2. Investigation of Real Samples

For functionality test, the CGO/PEDOT:PSS/Au/μ-Chip sensor was used to detect 4-CP from the industrial ETP plant water (S1) and household wastewater (S2). We initially remove the solid particles from the wastewater by filtration. Then, these real samples were analyzed using an electrochemical method by the developed CGO/PEDOT:PSS/Au/μ-Chip sensor probe as a WE. To this end, we employed the standard addition method in an aqueous medium to validate the correctness of 4-CP detection. Herein, 25 µl of aqueous 4-CP of various concentrations and an equal volume of real samples were mixed separately and studied in PBS by CGO/PEDOT:PSS/Au/μ-Chip sensor. Table 1 displays the outcomes obtained that showed that CGO/PEDOT:PSS/Au/μ-Chip assembly had a 4-CP recovery of ~100 percent. Therefore, we can conclude that this CGO/PEDOT:PSS/Au/μ-Chip sensor is acceptable, accurate, and reliable in determining 4-CP in real samples.

Electrochemical responses in 4-CP detection depend primarily on the surface morphology of doped nanocomposite material. If the surface of the CGO nanofiber touches the target 4-CP analyte, there is surface-mediated oxidation reaction occur. The 4-CP releases electron to the conduction band of prepared doped nanostructure material, so CGO, which ultimately increases the CGO/PEDOT:PSS/Au/μ-Chip sensor probe’s conductance, causes the increasing of sensor response. The electrochemical response is also consequently rising after each 4-CP injected analyte in the electrochemical solution. These processes increased the carrier concentration and consequently reduced the resistance on exposure to reducing liquids/analytes. At the room condition, the exposure of CGO/PEDOT:PSS/Au/μ-Chip surface to oxidize liquid/analytes results in a surface mediated process. The oxidize analyte 4-CP donates electrons to CGO/PEDOT:PSS/Au/μ-Chip surface. Therefore, resistance is decreased, and resultant conductance is increased. This causes the analyte response (current response) to increase with increasing potential, thus producing an electron supply to rapidly enhance conductance of the large surface area of CGO/PEDOT:PSS/Au/μ-Chip. The higher sensitivity of CGO/PEDOT:PSS coated chip could attribute to the good absorption (porous surfaces fabricated with coating), adsorption ability, high catalytic activity and good bio-compatibility of the CGO/PEDOT:PSS/Au/μ-Chip. The CGO/PEDOT:PSS/Au/μ-Chip assembly exhibits very high sensitivity towards 4-CP and extremely lower LOD than other 4-CP sensors, which is presented in Table 2 [13,20,21]. The CGO/PEDOT:PSS/Au/μ-Chip sensor is showed an excellent stability and reliability as well. 

The main features of the proposed CGO/PEDOT:PSS/Au/μ-Chip sensor is long-term stability, showing the enhanced electrocatalytic property in detecting 4-CP, having a useful nature, good reproducibility, wide LDR, high sensitivity, and low detection limit. Therefore, the CGO/PEDOT:PSS/Au/μ-Chip electrode showed exceptionally active electron-facilitating behavior in 4-CP detection.

## 4. Conclusions

We successfully synthesized and characterized the copper-doped gadolinium oxide (CGO) to modify a gold-coated µ-Chip for the development of an effective 4-CP sensor by electrochemical approach. Here, CGO/PEDOT:PSS/Au/μ-Chip electrode was successfully employed in determining aqueous 4-CP at ambient conditions. The modified CGO/PEDOT:PSS/Au/μ-Chip-based 4-CP chemical sensor exhibited an efficient electron-mediator during the 4-CP oxidation in PBS. The proposed 4-CP sensor showed high sensitivity, ultra-low LOD with a wide LDR in a short response time. The electrochemical approach validated the fabricated CGO/PEDOT:PSS/Au/μ-Chip sensor with various environmental samples including industrial effluent-water and waste-water and obtained reasonable performance. Finally, a new route to the development of an efficient electrochemical sensor is introduced by doped nanostructured materials embedded tiny micro-devices for the safety of the healthcare and environmental section.

## Figures and Tables

**Table 1:** Investigation of environmental samples by the CGO/PEDOT:PSS/Au/μ-Chip assembly.

Sample	4-CP Conc. Added	4-CP Conc. Determined by CGO/Au/µ-Chip	Recovery (%)	RSD (%) (*n* = 3)
S1	2.000 nM	2.060 nM	102.0	3.8
	2.000 µM	2.102 µM	105.7	4.4
S2	2.000 nM	1.984 nM	97.1	3.6
	2.000 µM	1.930 µM	96.0	4.3

**Table 2:** Comparison of different electrochemical processes for specific 4-CP detection.

Material	Method	LDR (μM)	LOD (μM)	Sensitivity (μAμM^−1^cm^−2^)	Refs.
rGO/MFO/SPCE	DPV	0.001 to 700	0.0012	6.836	[13]
Bi_2_MoO_6_	DPV	0.1–39.1 & 46.6–110.1	0.008297 & 0.01097	0.0287 μAμM^−1^	[21]
MWCNTs	CE	10–50 μg/L.	0.32 μg	-	[20]
CGO/PEDOT: PSS/Au/μ-Chip	I–V	1.0 nM–1.0 mM	1.3 ± 0.1 pM	2.4214	This work

Bi_2_MoO_6_ = Bismuth molybdate; MWCNTs = Multi-Walled Carbon Nanotubes; CE = Capillary Electrophoresis; rGO = Reduced Graphene Oxide; MFO = Magnesium Ferrites (MgFe_2_O_4_); SPCE = Screen-Printed Carbon paste Electrode.

## Data Availability

Not Applicable.

## References

[1] Forryan C.L., Lawrence N.S., Rees N.V., Compton R.G. (2004). Voltammetric Characterisation of the Radical Anions of 4-Nitrophenol, 2-Cyanophenol and 4-Cyanophenol in *N*,*N*-Dimethylformamide Electrogenerated at Gold Electrodes. J. Electroanal. Chem..

[2] DellaGreca M., Monaco P., Pinto G., Pollio A., Previtera L., Temussi F. (2001). Phytotoxicity of Low-Molecular-Weight Phenols from Olive Mill Waste Waters. Bull. Environ. Contam. Toxicol..

[3] Dean-Ross D., Rahimi M. (1995). Toxicity of Phenolic Compounds to Sediment Bacteria. Bull. Environ. Contam. Toxicol..

[4] Michałowicz J., Duda W. (2007). Phenols-Sources and Toxicity. Pol. J. Environ. Stud..

[5] Schweigert N., Zehnder A.J.B., Eggen R.I.L. (2001). Chemical Properties of Catechols and Their Molecular Modes of Toxic Action in Cells, from Microorganisms to Mammals. Environ. Microbiol..

[6] Hirose M., Takesada Y., Tanaka H., Tamano S., Kato T., Shirai T. (1997). Carcinogenicity of antioxidants BHA, caffeic acid, sesamol, 4-methoxyphenol and catechol at low doses, either alone or in combination, and modulation of their effects in a rat medium-term multi-organ carcinogenesis model. Carcinogenesis.

[7] Przybylski P., Wojciechowski G., Brzezinski B., Zundel G., Bartl F. (2003). FTIR Studies of the Interactions of 1,3,5-Triazabicyclo [4.4.0] Dec-5-Ene with 4-Tert-Butylphenol and 4-Cyanophenol. J. Mol. Struct..

[8] Romonchuk W.J., Bunge A.L. (2010). Mechanism of Enhanced Dermal Permeation of 4-Cyanophenol and Methyl Paraben from Saturated Aqueous Solutions Containing Both Solutes. Skin Pharmacol. Physiol..

[9] Medendorp J., Yedluri J., Hammell D.C., Ji T., Lodder R.A., Stinchcomb A.L. (2006). Near-Infrared Spectrometry for the Quantification of Dermal Absorption of Econazole Nitrate and 4-Cyanophenol. Pharm. Res..

[10] Bronner C., Wenger O.S. (2012). Proton-Coupled Electron Transfer between 4-Cyanophenol and Photoexcited Rhenium(I) Complexes with Different Protonatable Sites. Inorg. Chem..

[11] Fukuto T.R., Metcalf R.L. (1956). Pesticidal Activity and Structure, Structure and Insecticidal Activity of Some Diethyl Substituted Phenyl Phosphates. J. Agric. Food Chem..

[12] Adamska G., Dabrowski R., Dziabuszek J. (1981). A Convenient Method of Obtaining 2-Cyano-4-Alkylphenols, 4-Cyanophenol and 4-Cyanoaniline. Mol. Cryst. Liq. Cryst..

[13] Hwa K.Y., Ganguly A., Tata S.K.S. (2020). Influence of Temperature Variation on Spinel-Structure MgFe2O4 Anchored on Reduced Graphene Oxide for Electrochemical Detection of 4-Cyanophenol. Microchim. Acta.

[14] Binev Y.I. (2001). Ab Initio MO and Experimental Studies on the Vibrational Spectra and Structure of 4-Hydroxybenzonitrile and of Its Anion. J. Mol. Struct..

[15] Dimitrova Y., Tsenov J.A. (2007). Theoretical Study of the Structures and Vibrational Spectra of the Hydrogen-Bonded Systems of 4-Cyanophenol with N-Bases. Spectrochim. Acta Part A Mol. Biomol. Spectrosc..

[16] Gomes J.R.B., Liebman J.F., Da Silva M.A.V.R. (2007). The Thermodynamics of the Isomerization of Cyanophenol and Cyanothiophenol Compounds. Struct. Chem..

[17] Forryan C.L., Compton R.G. (2003). Studies of the Electrochemical Reduction of 4-Nitrophenol in Dimethylformamide: Evidence for a Change in Mechanism with Temperature. Phys. Chem. Chem. Phys..

[18] Bols N.C., Boliska S.A., Dixon D.G., Hodson P.V., Kaiser K.L.E. (1985). The Use of Fish Cell Cultures as an Indication of Contaminant Toxicity to Fish. Aquat. Toxicol..

[19] Hamai S., Satoh N. (1997). Inclusion Effects of Cyclomaltohexa- and Heptaose (α- and β-Cyclodextrins) on the Acidities of Several Phenol Derivatives. Carbohydr. Res..

[20] Alothman Z.A., Badjah A.Y., Locatelli M. (2020). Multi-Walled Carbon Nanotubes Solid-Phase Extraction and Capillary Electrophoresis Methods for the Analysis of 4-Cyanophenol and 3-Nitrophenol in Water. Molecules.

[21] Jesila J.A., Umesh N.M., Wang S.F., Mani G., Alothman A.A., Alshgari R.A. (2021). An Electrochemical Sensing of Phenolic Derivative 4-Cyanophenol in Environmental Water Using a Facile-Constructed Aurivillius-Structured Bi_2_MoO_6_. Ecotoxicol. Environ. Saf..

[22] Ahmed J., Rashed A., Faisal M., Harraz F.A., Jalalah M., Alsareii A. (2021). Applied Surface Science Novel SWCNTs-Mesoporous Silicon Nanocomposite as Efficient Non-Enzymatic Glucose Biosensor. Appl. Surf. Sci..

[23] Rahman M.M., Ahmed J., Asiri A.M. (2017). Development of Creatine Sensor Based on Antimony-Doped Tin Oxide (ATO) Nanoparticles. Sens. Actuators B Chem..

[24] Ahmed J., Rahman M.M., Siddiquey I.A., Asiri A.M., Hasnat M.A. (2017). Efficient Bisphenol-A Detection Based on the Ternary Metal Oxide (TMO) Composite by Electrochemical Approaches. Electrochim. Acta.

[25] Ahmed J., Rahman M.M., Siddiquey I.A., Asiri A.M., Hasnat M.A. (2018). Efficient Hydroquinone Sensor Based on Zinc, Strontium and Nickel Based Ternary Metal Oxide (TMO) Composites by Differential Pulse Voltammetry. Sens. Actuators B Chem..

[26] Rahman M.M., Ahmed J. (2018). Cd-Doped Sb_2_O_4_ Nanostructures Modified Glassy Carbon Electrode for Efficient Detection of Melamine by Electrochemical Approach. Biosens. Bioelectron..

[27] Rahman M.M., Ahmed J., Asiri A.M., Alamry K.A. (2020). Fabrication of a Hydrazine Chemical Sensor Based on Facile Synthesis of Doped NZO Nanostructure Materials. New J. Chem..

[28] Subhan M.A., Chandra Saha P., Ahmed J., Asiri A.M., Al-Mamun M., Rahman M.M. (2020). Development of an Ultra-Sensitive Para-Nitrophenol Sensor Using Tri-Metallic Oxide MoO_2_ Fe_3_O_4_ CuO Nanocomposites. Mater. Adv..

[29] Subhan M.A., Saha P.C., Sumon S.A., Ahmed J., Asiri A.M., Rahman M.M., Al-Mamun M. (2018). Enhanced Photocatalytic Activity and Ultra-Sensitive Benzaldehyde Sensing Performance of a SnO_2_·ZnO·TiO_2_ Nanomaterial. RSC Adv..

[30] Katowah D.F., Hussein M.A., Alam M.M., Ismail S.H., Osman O.I., Sobahi T.R., Asiri A.M., Ahmed J., Rahman M.M. (2020). Designed Network of Ternary Core-Shell PPCOT/NiFe_2_O_4_/C-SWCNTs Nanocomposites. A Selective Fe^3+^ Ionic Sensor. J. Alloys Compd..

[31] Rahman M.M., Ahmed J., Asiri A.M., Siddiquey I.A., Hasnat M.A. (2016). Development of Highly-Sensitive Hydrazine Sensor Based on Facile CoS_2_-CNT Nanocomposites. RSC Adv..

[32] Rahman M.M., Ahmed J., Asiri A.M., Siddiquey I.A., Hasnat M.A. (2016). Development of 4-Methoxyphenol Chemical Sensor Based on NiS_2_-CNT Nanocomposites. J. Taiwan Inst. Chem. Eng..

[33] Safavi A., Maleki N., Moradlou O. (2008). A Selective and Sensitive Method for Simultaneous Determination of Traces of Paracetamol and P-Aminophenol in Pharmaceuticals Using Carbon Ionic Liquid Electrode. Electroanalysis.

[34] Huang W., Hu W., Song J. (2003). Adsorptive Stripping Voltammetric Determination of 4-Aminophenol at a Single-Wall Carbon Nanotubes Film Coated Electrode. Talanta.

[35] Rahman M.M., Khan S.B., Jamal A., Faisal M., Asiri A.M. (2012). Highly Sensitive Methanol Chemical Sensor Based on Undoped Silver Oxide Nanoparticles Prepared by a Solution Method. Microchim. Acta.

[36] Maalej N.M., Qurashi A., Assadi A.A., Maalej R., Shaikh M.N., Ilyas M., Gondal M.A. (2015). Synthesis of Gd_2_O_3_:Eu Nanoplatelets for MRI and Fluorescence Imaging. Nanoscale Res. Lett..

[37] Singh S.K., Kumar K., Rai S.B. (2009). Multifunctional Er^3+^-Yb^3+^ Codoped Gd_2_O_3_ Nanocrystalline Phosphor Synthesized through Optimized Combustion Route. Appl. Phys. B Lasers Opt..

[38] Hirai T., Orikoshi T. (2004). Preparation of Gd_2_O_3_:Yb,Er and Gd_2_O_2_S:Yb,Er Infrared-to-Visible Conversion Phosphor Ultrafine Particles Using an Emulsion Liquid Membrane System. J. Colloid Interface Sci..

[39] Pandey A., Kroon R.E., Swart H.C. Fluorescence Behaviour of Eu Doped Gd_2_O_3_ Nanosheets via CuO Incorporation. Proceedings of the SAIP Conference.

[40] Majeed S., Shivashankar S.A. (2014). Novel Spherical Hierarchical Structures of GdOOH and Eu:GdOOH: Rapid Microwave-Assisted Synthesis through Self-Assembly, Thermal Conversion to Oxides, and Optical Studies. J. Mater. Chem. C.

[41] Majeed S., Shivashankar S.A. (2014). Rapid, Microwave-Assisted Synthesis of Gd_2_O_3_ and Eu:Gd_2_O_3_ Nanocrystals: Characterization, Magnetic, Optical and Biological Studies. J. Mater. Chem. B.

[42] Atabaev T.S., Lee J.H., Han D.W., Kim H.K., Hwang Y.H. (2015). Fabrication of Carbon Coated Gadolinia Particles for Dual-Mode Magnetic Resonance and Fluorescence Imaging. J. Adv. Ceram..

[43] Delice S., Isik M., Gasanly N.M. (2018). Characterization of Trap Centers in Gd_2_O_3_ Nanoparticles by Low Temperature Thermoluminescence Measurements. Optik.

[44] Chinthakuntla A., Rao K.V., Ashok C., Rao K., Chakra C.S. (2014). Structural Analysis of CuO Nanomaterials Prepared by Novel Microwave Assisted Method. J. Atoms Mol..

[45] Shi L.B., Tang P.F., Zhang W., Zhao Y.P., Zhang L.C., Zhang H. (2017). Green Synthesis of CuO Nanoparticles Using Cassia Auriculata Leaf Extract and in Vitro Evaluation of Their Biocompatibility with Rheumatoid Arthritis Macrophages (RAW 264.7). Trop. J. Pharm. Res..

[46] Mandal M., Nagaraj R., Chattopadhyay K., Chakraborty M., Chatterjee S., Ghosh D., Bhattacharya S.K. (2021). A High-Performance Pseudocapacitive Electrode Based on CuO–MnO_2_ Composite in Redox-Mediated Electrolyte. J. Mater. Sci..

[47] Azam A., Ahmed A.S., Oves M., Khan M.S., Memic A. (2012). Size-Dependent Antimicrobial Properties of CuO Nanoparticles against Gram-Positive and -Negative Bacterial Strains. Int. J. Nanomed..

[48] Liu H., Liu J. (2016). Hollow Mesoporous Gd_2_O_3_:Eu^3+^ Spheres with Enhanced Luminescence and Their Drug Releasing Behavior. RSC Adv..

[49] Gai S., Yang P., Wang D., Li C., Niu N., He F., Li X. (2011). Monodisperse Gd_2_O_3_:Ln (Ln = Eu^3+^, Tb^3+^, Dy^3+^, Sm^3+^, Yb^3+^/Er^3+^, Yb^3+^/Tm^3+^, and Yb^3+^/Ho^3+^) Nanocrystals with Tunable Size and Multicolor Luminescent Properties. CrystEngComm.

[50] Tanvir N.B., Yurchenko O., Wilbertz C., Urban G. (2016). Investigation of CO_2_ Reaction with Copper Oxide Nanoparticles for Room Temperature Gas Sensing. J. Mater. Chem. A.

